# A hypothetic model for examining the relationship between happiness, forgiveness, emotional reactivity and emotional security

**DOI:** 10.1007/s12144-022-02909-2

**Published:** 2022-03-29

**Authors:** Mustafa Ercengiz, Serdar Safalı, Alican Kaya, Mehmet Emin Turan

**Affiliations:** Faculty of Education, Agri Ibrahim Cecen University, Agri, Turkey

**Keywords:** Happiness, Forgiveness, Emotional reactivity, Emotional security

## Abstract

The ultimate goal of life is happiness, according to Plato. Perhaps the most critical questions in the life of human beings have been on happiness and processes that affect happiness. The present study was planned during the COVID-19 pandemic; perhaps human beings are most needed for happiness. The original hypothetical model and the findings constitute the powerful and different aspects of the present study. This study determined a hypothetical model to examine the relationships among happiness, forgiveness, emotional reactivity, and emotional security. The participant group of the study consists of a total of 916 individuals from Turkey, 617 women, and 299 men. The age scale of the participants is between 18-25. Participants completed the Heartland Forgiveness Scale, the Emotional Security Scale, the Emotional Reactivity Scale, and the Oxford Happiness Scale. Mediation analysis was conducted using Hayes’ (2017) process macro. According to the proposed model in the study, emotional reactivity mediates the relationship between forgiveness and happiness. As the individual’s forgiveness increases, their emotional reactivity decreases, and as the emotional reactivity decreases, the individual’s level of happiness increases.

As social beings, humans have the potential to communicate and maintain communication with other individuals (Tanhan, 2020; Yedirir & Hamarta, 2015). Emotions can be expressed explicitly or implicitly to enable this communication (Chung et al., 2021). Emotions are vital in various situations, such as creating meaning (Immordino-Yang et al., 2016), learning (Hill et al., 2021), memory (Montijn et al., 2021), motivation (Cromwell et al., 2020), life satisfaction (Turan, 2021) and decision making (Gosling et al., 2020). Exposure to emotions enables people who experience emotions to reflect that emotion with the help of neuron structures such as mirror neurons or transform the experienced emotion into a concrete form (learning) (Fabbri-Destro & Rizzolatti, 2008; Singer & Lamm, 2009). Therefore, learning has an essential role in the emergence of emotions (Shablack et al., 2020; Snefjella et al., 2020). Although emotions are vital in all human life, researchers have attached great importance to their first childhood experiences in emotions and emotional development (Bronfenbrenner, 1986; Niklas et al., 2016; Rodriguez & Tamis‐LeMonda, 2011).

Early childhood experiences are crucial in individuals’ personality development and subjective well-being (Bowlby, 1951; Uddin et al., 2020). The health of first childhood life leads to a sense of trust in individuals, while the opposite leads to the emergence of insecurity (Davies et al., 2002; Hoyniak et al., 2020). In addition, in the emotional security theory, it has been stated that individuals may experience prolonged, intense emotional states such as stress, anxiety, and behavioral or emotional disorders as a result of the feeling of insecurity arising from family relationships within the family system (Davies & Cummings, 1994; Girme et al., 2020). The emotional security state in which emotional intensity and disorder are seen can cause emotional reactivity, which can also be associated with the emotional disorder (Cheung, 2020; Davies et al., 2020; Nock et al., 2008).

Emotional reactivity refers to the situation where it takes a long time for the individual to regain their previous emotional level after strong and intense emotional experiences (Davidson, 1998; Nock et al., 2008). Emotional reactivity can also be associated with many psychopathological conditions such as anxiety disorder, depression, and personality disorders (Bylsma et al., 2011; Carthy et al., 2010; Fox et al., 2010). Thus, it can be said that individuals who experience intense emotions have more behavioral problems and are prone to emotionally negative emotions (Silk et al., 2003). In this context, it can be stated that individuals who can control their emotional reactivity level may experience more positive emotions (Bylsma et al., 2011). Happiness is considered one of the basic positive emotions (Joshanloo, 2014; Santos & Egerstedt, 2020).

Happiness is a condition for many people to have a positive and beautiful life (Brülde, 2007; King & Napa, 1998; O’neill, 2008). It can be expressed that happy people have positive experiences and these positive experiences turn into personality traits (Deneve & Cooper, 1998; Ford et al., 2016; Pelechano et al., 2013). Previous research on happiness revealed that happiness is associated with positive experiences such as higher physical health, higher life satisfaction, longer life, and improvements in social relationships (Diener & Seligman, 2002; Graham, 2008). Studies on happiness also emphasize the relationship between happiness and forgiveness (Sudirman et al., 2019).

It can be thought that forgiveness improves interpersonal relationships and increases an individual’s level of subjective well-being (Bono et al., 2008; Johnson et al., 2013). The attack of revenge and retaliation is reduced by forgiveness when cognitive control resources are used, which can help resolve conflicts (Fincham et al., 2007; Wilkowski et al., 2010). Inability to forgive is associated with stress-related poor mental health, rumination, and depression (Ermer and Proulx, 2016; Griffin et al., 2015).

## Forgiveness, Happiness, and Emotional Reactivity

Forgiveness can generally be defined as avoiding the event or person that caused this negative situation after a negative situation (transgression) occurs, decreasing motivation for revenge, and replacing negative emotions with positive ones (McCullough et al*.,* 1998; Worthington et al., 2005). In this respect, forgiveness functions as a coping strategy that helps to replace negative situations with positive ones (Gall & Bilodeau, 2020; Oti-Boadi et al., 2020). Forgiveness is a resource related to many positive situations enabling the emergence of a beautiful life by strengthening the interaction between people (Karremans et al., 2005; Van der Wal et al., 2017). Considering the definition of forgiveness of replacing negative emotions with positive emotions, the consequences of these positive emotions will increase the individual’s subjective well-being and increase the level of happiness (Russell, 2020).

Happiness is associated with the intensity of emotional components (Diener & Seligman, 2002) and many positive situations like well-being (Michalos, 2008). On the other hand, the intensity of affect and unhappiness associated with negative situations such as mania, depressive mood, and depression (Gruber et al., 2011; Konjedi & Maleeh, 2017; Park et al., 2017). Considering that the emotional reactivity state is related to the intensity of emotions (Nock et al., 2008), it can be considered one of the negative situations that can be experienced.

Emotional reactivity is related to emotions’ sensitivity, continuity, and intensity (Nock et al., 2008). Emotions’ rapid changes and emotional regulation problems cause multiple maladaptation problems and psychopathologies (Millgram et al., 2020; Zafar et al., 2021). People with high emotional reactivity are at risk of experiencing emotionally negative situations more (Buehler & Welsh, 2009; Maremmani et al., 2018). On the other hand, individuals with low emotional reactivity have a protective effect against the risk of experiencing negative experiences (Bartle-Haring et al., 2002; McLaughlin et al., 2010).

- *H1. Forgiveness is related to happiness.*
- *H2. Emotional reactivity mediates the relationship between forgiveness and happiness*.

## Emotional Security, Forgiveness, and Happiness

Emotional security plays an essential role in the health and behavioral aspects of early childhood experiences within the family in early adulthood and beyond (Dorn & Schudlich, 2020). In addition to the feeling of security in the family, which is considered very important in the child’s development, if family members witnesses conflict in the family atmosphere, this may cause the child to feel emotionally insecure, especially (Cantón-Cortés et al., 2019; El-Sheikh et al., 2007). As the experiences of family conflicts and conflicts that the child witnesses in the family become permanent, the emotional insecurity of the child in later periods becomes ingrained (Li et al., 2020). This situation may cause many psychopathologies in other stages of the developmental period (Liese et al., 2020; Liu et al., 2020; Nandrino et al., 2020). On the contrary, children who gain trust in the family experience positive life patterns and positive moods in later periods (Hoffman et al., 2017). Moreover, increasing emotional confidence in the family reduces the effects of many negative situations such as conflict and suicidal thoughts (Cantón-Cortés et al., 2020; Goeke-Morey et al., 2007). It also contributes to increasing more positive experiences and affects (attention, self-esteem, social competence) (Commodari, 2013; Gross et al., 2017). It can be claimed that forgiveness is one of these positive experiences (Lawler-Row et al., 2006).

Forgiveness is a process that allows the individual to reduce negative emotions and behaviors such as anger and aggression. In this direction, the individual can have neutral or positive feelings towards the individual with whom they have negative emotions (Babin et al., 2021; McCullough et al., 1997). This process allows for reducing aggression or anger towards a transgressor and more positive feelings, thoughts, and behaviors towards that person (Knutson et al., 2008). In this context, it can be emphasized that the social value of forgiveness is vital for the maintenance and development of human relations (La Fors, 2020). In addition to its social dimension, the positive effects of forgiveness on individuals’ lives (physical health, subjective well-being, happiness) are known (Long et al., 2020).

Happiness, one of the positive effects on individual lives, consists of three dimensions. These are dimensions of having a positive emotion, being connected to life, and meaning in life (Seligman, 2002). Having positive emotions means that individuals have positive emotions about the past, present, future and gain the skills necessary to experience these emotions intensely. Studies have shown that these favorable situations have positive relationships among senses of security, happiness, and forgiveness (Belicki et al., 2020; Ercengiz, 2019). Emotional security is thought to play a mediating role between forgiveness and happiness in this context.

- H3. Emotional security mediates the relationship between forgiveness and happiness.

## Emotional Security, Emotional Reactivity, Forgiveness, and Happiness

The first place where the child’s sense of security is gained is the family environment (Dansby Olufowote et al., 2019). Witnessing conflicts in family relationships between parents is devastating, especially in early childhood (Black, 2020). Early childhood experiences form the basis of individual’s relationships with others (Masterson, 2008). Researchers stated that changes in adulthood personality patterns might be related to childhood experiences (Eisenberg et al., 2014; Fletcher & Schurer, 2017). Children who gain emotional confidence in the family system during childhood may be happier and less prone to experiencing psychological problems thanks to a safe and supportive environment and gaining positive qualities such as empathy (Shoshani et al., 2021) and self-esteem (Pan et al., 2018). It was found that having problems gaining emotional confidence was associated with negative situations such as aggression and anxiety (Bergman et al., 2014) and depression (Cummings et al., 2013).

One of these adverse situations is emotional reactivity (Aki et al., 2020; Silva et al., 2016). Emotional reactivity includes strong and intense responses to a range of stimuli and emphasizes the extent of emotions experienced by an individual before reverting for a long time (Nock et al., 2008). It is associated with or predictive of many psychopathological conditions such as anxiety, stress (Ripper et al., 2018), suicidal thoughts, and suicide (Shapero et al., 2019). In addition, emotional reactivity can be considered a very stable personality trait with biological aspects (Berenbaum & Williams, 1995; Gottlieb, 1983). It is also a component of emotional dysregulation and can contribute to "emotional output” (Gross & Thompson, 2007). In this context, it can be explained that it is related to forgiveness, which has an emotional aspect and can be considered an emotional output (Chagigiorgis & Paivio, 2008).

Forgiveness, which is considered necessary in replacing adverse reactions such as anger, aggressive thoughts, disappointment with positive results, has a solid and healing effect for the forgiving person (Tekinalp & Terzi, 2012). In addition, it improves the interpersonal relationships of the forgiving, and it is beneficial in developing healthy relationships (Arfasa & Weldmeskel, 2020; Watkins et al., 2011) and functions as a coping mechanism in stressful situations (McCullough, 2000). Forgiveness is seen in the literature to be associated with favorable situations and emotions (happiness, resilience, gratitude) (Maltby et al., 2005; Nagra et al., 2016).

Happiness, one of these positive emotions, has attracted the attention of researchers for many years, considering its effect on people’s longer lives and health (Lozano & Solé-Auró, 2021). However, there is no definite standard definition in the literature (Ryan & Deci, 2001). Happiness means a generally positive mood, a global assessment of life satisfaction, living a good life, or reasons that make people happy by being interpreted according to context (Diener, 2006). Happiness is associated with two components, such as content and context (Delle Fave et al., 2011). The family atmosphere is one of the essential variables among the factors associated with happiness (Fernandes et al., 2020). Relationships between couples in the family can cause various emotions such as satisfaction, happiness, unhappiness, and trust to be experienced within the family (Yedirir & Hamarta, 2015). The continuity and intensity of these affects vary from individual to individual (Lucas & Baird, 2004). In addition to the fact that these differences can be associated with family relationships, the incompatibilities in these relationships can cause individuals to intensify their emotional reactions. It is thought that this situation may cause emotional reactivity.

- H4. Emotional reactivity and emotional security serially mediate the relationship between forgiveness and happiness.

## The Purpose, Importance, and Theoretical Basis of the Study

The study proposed a hypothetical model for understanding the interaction patterns of emotional security, forgiveness, happiness, and emotional reactivity in individuals’ lives. Considering the ages and life roles of the university students, who constitute the sample group in the study, it can be claimed that the study group consists of individuals in pre-adulthood. The pre-adulthood period, in addition to rapid psychological development (Tanhan et al., 2021; Viner, 2015), also constitutes a risk group for various unfavorable conditions such as anxiety, depression, nutritional disorders, and stress (Ponce-Pardo et al., 2021; Winpenny et al., 2020). In addition, this period is a peak where people with various disorders or undesirable life patterns begin to experience increases in various mood disorders and psychotic disorders (Scott et al., 2021). According to adulthood theory (Arnett, 2000), it represents a crucial stage of development in which essential skills and experiences are acquired in young adults’ achieving various adult roles. Thus, considering the specified characteristics of the pre-adult period, it can be said that understanding the interactional patterns of the emotional structures of individuals in this period is essential. This present study is important due to the focus of pre-adulthood experiences on happiness. The pre-adulthood period is one of the crucial period related to happiness. In addition, Covid-19 has been reported to significantly lower the level of happiness (Yıldırım & Güler, 2021). When the low level of happiness is considered their relationship with undesirable situations during the pre-adulthood period, it is important for the study of the pre-adulthood period, which is reported that the level of happiness falls significantly in the Covid-19 period.

**Figure 1:**
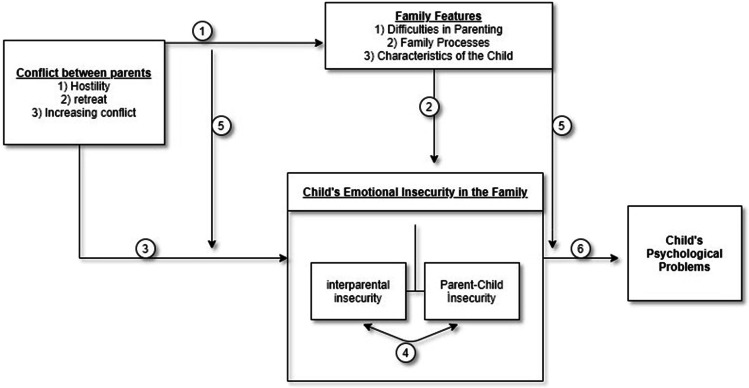
Emotional security theory model (Davies & Woitach, 2008)

In the study, the approaches of dynamic developmental theorists (Melaine Klein, Margaret Mahler, Otto Kernber, and John Masterson) regarding early childhood experiences have been a crucial reference point in the construction of a hypothetical model related to emotional security, emotional reactivity, happiness and forgiveness of individuals in pre-adulthood. In addition, the proposed hypothetical model has been constructed concerning dynamic developmental approaches. Dynamic approaches focus on interactional patterns and causes of behaviors rather than results of individuals’ behaviors is an essential parameter in constructing the research according to dynamic approaches. Therefore, the hypothetical model proposition of the interactional pattern of the causes is considered important in the development of the outcomes instead of the outcomes of the behavior of the individuals is the primary goal (Fig. 1).

## Method

### Participants and Procedure

The participant group of the study consists of 916 people from Turkey, 617 women, and 299 men. The age range of the participants is between 18-25. The convenience sampling method was used to determine the participants. The convenience sampling method is to select the sample from easily accessible units due to time, money, and labor limitations (Büyüköztürk et al., 2014). All data were collected from university students from Turkey via Google forms. During the application of the measurement tools, there was a voluntary basis. In order to prevent trust problems that may occur while answering the questions on the scales, it was requested that personal information not be written into the scale batteries. Mediation Analysis was conducted using Hayes’ (2017) Process Macro.

### Measures

#### The Heartland Forgiveness Scale

The Heartland Forgiveness Scale, developed by Thompson et al. (2005) and adapted to Turkish by Bugay and Demir (2010), is rated as 7-point Likert, and consists of 18 items and 3 sub-dimensions, respectively, forgiveness of self, forgiveness of others, and forgiveness of the situations. In this study, Cronbach’s alpha reliability values for sub-dimensions of the scale were calculated as .64, .79, and .76 for forgiveness of self, forgiveness of others, and the forgiveness of situations, respectively. The Cronbach’s alpha coefficient for the scale’s total score was found to be .81.

#### Emotional Security Scale

Davies et al. (2002) developed the emotional security scale to evaluate the perception of emotional security in children aged 11-15. The scale was adapted to Turkish by Sakız (2011). The validity and reliability studies of the version of the scale developed to evaluate the emotional security perception of university students were conducted by Şendil (2016). The scale is rated as 4-point Likert, and consists of 24 items and 6 sub-dimensions: constructive family representations, destructive family representations, spread of conflict, avoidance, emotional arousal, and difficulties in emotional regulation. Internal consistency coefficients of the subscales were calculated between .68 and .88.

#### Oxford Happiness Questionnaire Short Form

Oxford Happiness Questionnaire Short Form (OHQ-SF) is a 5-point Likert-type scale developed by Hills and Argyle (2002) to evaluate the level of happiness and adapted to Turkish by Doğan and Akıncı Çötok (2011). The scale consists of 7 items and one dimension. The internal consistency and test-retest reliability coefficients were determined as .74, and .85, respectively. As a result, it can be said that OHQ-SF is a valid and reliable measurement tool that can be used to measure the happiness of Turkish university students.

#### The Emotion Reactivity Scale

The Emotion Reactivity Scale, developed by Nock et al. (2008) and adapted to Turkish by Seçer et al. (2013), is a 4-point Likert-type scale and consists of 17 items and 3 sub-dimensions. It was found that the internal consistency coefficient to determine the reliability of the whole scale was .91, the sensitivity sub-dimension was .86, the emotional reactivity sub-dimension was .76, the psychological resilience sub-dimension was .81. It can be said that the scale had adequate internal consistency values. As a result, it can be said that the Emotion Reactivity Scale is a valid and reliable measurement tool that can be used to measure the emotional reactivity of university students.

### Ethics

Ethics committee approval for this study was obtained from Agri Ibrahim Cecen University Ethics Committee. In addition, every stage of the study was carried out in accordance with the Declaration of Helsinki.

### Data Analysis

The data was analyzed through the statistical package program. The assumptions were required to perform analyzes before the analyzes were tested. In this context, the kurtosis-skewness values of the data set were checked. It was determined that the normality and linear relationship assumptions, which are the prerequisites of parametric tests, were met. In addition, it was determined that the level of correlation between the variables was not high. Tolerance, VIF, and CI values were examined to understand that there is no multicollinearity problem. While the tolerance value should be less than .10, the VIF value should be less than 10, and the CI value should be between 10-30 (Albayrak, 2005). As a result of the study, no multicollinearity problem was determined. In order to determine the outliers, Mahalanobis distance values were examined, and it was determined that the data obtained from 25 participants had outliers. The determined data were not included in the analysis, and the analysis was carried out on 916 data.

**Figure 2:**
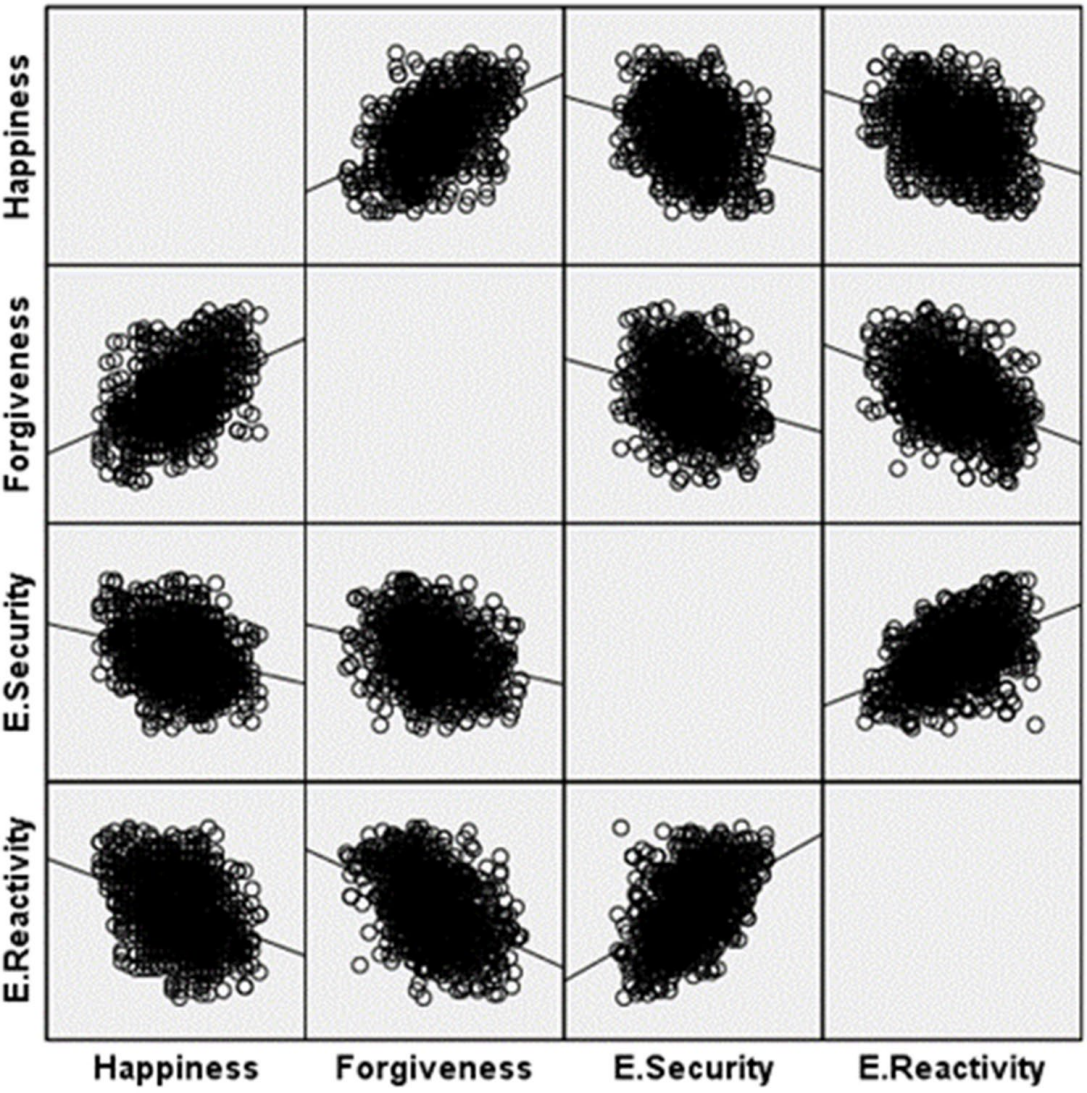
Scatter matrix for Research Variables

When the scatter matrix given in Figure 2 is examined, it is seen that there are no extreme outliers in the linear evaluation. The data show a normal distribution, as well as there are positive and negative dimensions of the correlations between happiness, forgiveness, emotional security, and emotional reactivity.

## Results

### Preliminary Analyses

Descriptive statistics, correlations, and reliabilities for the study variables are presented in Table 1. Emotional reactivity was positively associated with emotional security (*r =* .47, *p <* .001)and negatively associated with both forgiveness (r *=* −.42, p < .001), and happiness (r *=* − .35, p *<* .001). Emotional security was negatively associated with forgiveness (r *=* -.26, p *<* .001) and positively associated with happiness (r *=* .26, p *<* .001). Forgiveness was positively associated with happiness (r *=* .45, p *<* .001).

**Table 1:** Descriptive statistics and bivariate correlations among variables among the total sample (N = 916).

	E.R	E.S	F.R	H.P
E.R	-			
E.S	.47	-		
F.R	−.42	-.26	-	
H.P	-.35	.26	.45	-
Mean	45.73	59.49	79.62	22.88
Std. Deviation	9.78	9.74	14.33	4.53
Skewness	-.01	.09	.16	-.05
Kurtosis	-.65	-.30	-.12	-.44
Range	47	49	77	22

*p <.001. E.R = Emotional reactivity; E.S = Emotional security; F.R = Forgiveness; H.P = Happiness*

Note. Correlations between all main variables in the study are shown (Emotional reactivity, emotional security, forgiveness and happiness). Bivariate correlations showed that all variables were moderately related to each other.

## Serial Multiple Mediational Analyses - Modeling Data

Results of the serial mediation analyses are presented in Figure 2. It was found a direct effect of forgiveness on happiness (total effect, *β=* .453, *p <* .001). When the mediators (emotional reactivity and emotional security) were included, the analysis results showed that this coefficient was still significant (direct effect, *β =* .365, *p <* .001]. Forgiveness was also found to be a positive predictor of emotional reactivity (*β =* -.421, *p <* .001), and emotional security (*β =* -.071, *p <* .05). According to the results, Hypothesis 1 has been confirmed.

It was found a significant indirect effect of forgiveness on happiness via emotional reactivity (*indirect effect =* .06, SE = .02, *95%* CI = [.03, .09]). Also, the indirect effect of forgiveness on happiness via emotional security was also significant (*indirect effect =* .01, SE = .01, *95%* CI = [.01, .02]). According to results in the relationship between forgiveness and happiness, emotional reactivity and emotional security have mediating effects separately. Hypothesis 2 and hypothesis 3 have been confirmed.

Lastly, the indirect effects of forgiveness on happiness via both emotional reactivity and emotional security were tested. The relationship was significant with a point estimate of .02 (testing serial multiple mediation; SE = .01, *95%* CI = .03, .09). According to results in the relationship between forgiveness and happiness, emotional reactivity and emotional security have mediating effects serially. Hypothesis 4 has been confirmed.

**Figure 3:**
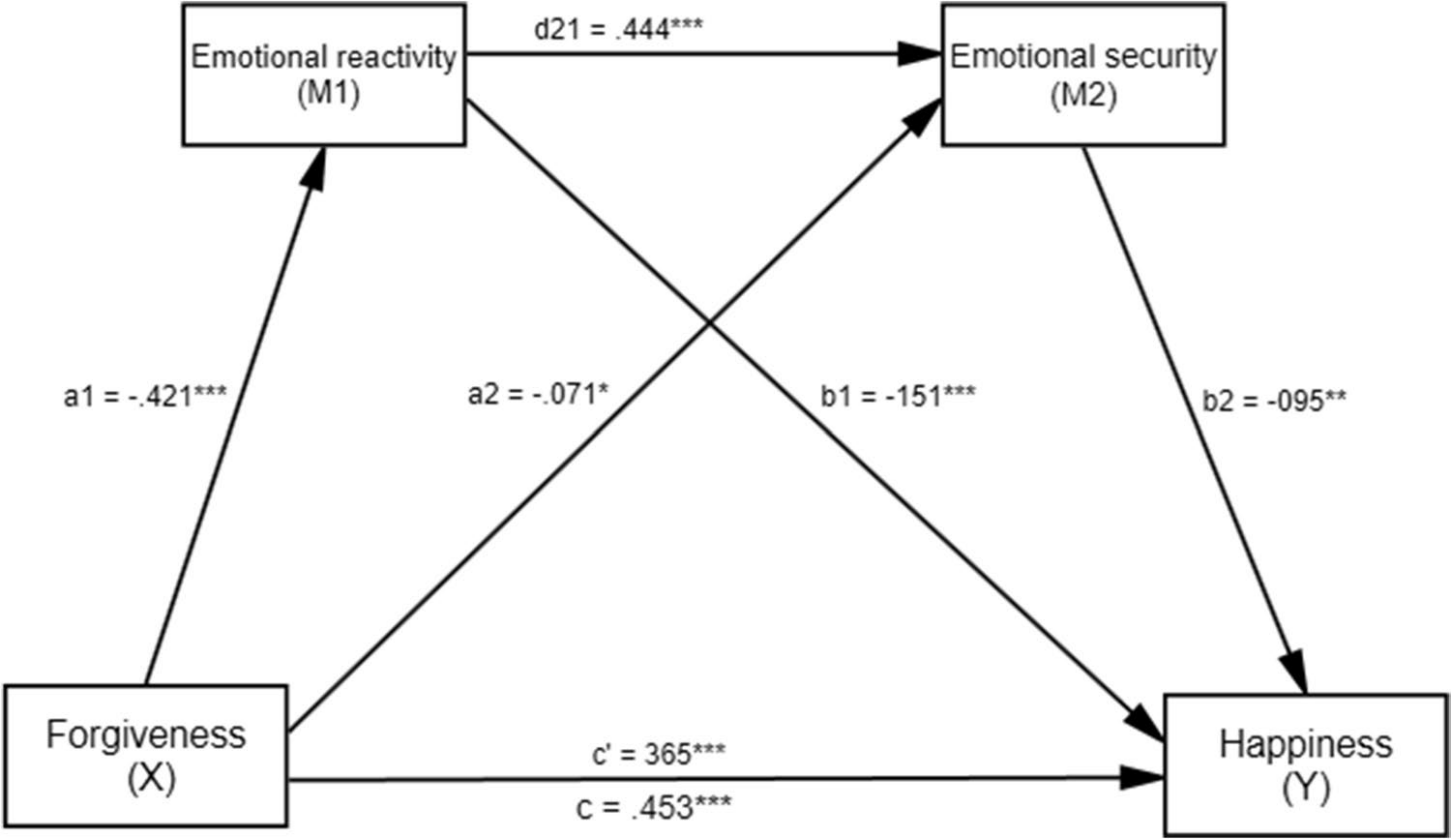
The results of serial multiple mediational mode.l Note. Y= Happiness, X=Forgiveness, M1 = Emotional reactivity, M2 = Emotional security

As a result, all hypotheses of the present study have been confirmed (see Table 2). The results have been indicated that forgiveness predicts happiness. It was found that there is an indirect relationship between forgiveness and happiness. The results showed that the relationship between forgiveness and happiness is partially mediated by emotional reactivity and emotional security (see Figure 3).

**Table 2.** Completely standardized indirect effect of forgiveness on happiness via emotional reactivity and emotional security

Path	Coefficient	95% CI	
		LL	UL
Forgiveness ➔ Emotional reactivity ➔ Happiness	.06	.03	.09
Forgiveness ➔ Emotional security ➔ Happiness	.01	.01	.02
Forgiveness ➔ Emotional reactivity ➔ Emotional security ➔ Happiness	.02	.01	.02
Total effect	.45	.12	.16
Direct effect	.36	.10	.14
Total indirect effect	.09	.06	.12

Note. CI = confidence interval, LL = lower limit, UL = upper limit

## Conclusion and Discussion

The general purpose of the study is to determine a hypothetical model to examine the relationship among happiness, forgiveness, emotional reactivity, and emotional security. According to the study’s findings, it was determined that emotional security mediates the relationship between forgiveness, and happiness. Individuals who feel emotionally secure, feel happier, forgive other individuals more and show less emotional reactivity because they show more conscious and positive behaviors.

In this context, firstly, the relationship between forgiveness and emotional reactivity was tested within the scope of the study. Findings showed that there is a significant relationship between forgiveness and emotional reactivity.

No studies have been found in which the findings obtained in the study can be compared. Although there is a positive correlation between forgiveness and self-compassion (Mansfield et al., 2015; Cleare et al., 2019; Wu et al., 2019) and a negative correlation between self-compassion and emotional reactivity (Leary et al., 2007; Mousavi Asl, et al., 2021), it can be said that there is a significant relationship between forgiveness and emotional reactivity. Therefore, within the scope of the research, it can be expressed that the significant relationship between forgiveness and emotional reactivity variables supports the literature data. It can be pointed out that there is a negative correlation between forgiveness and emotional reactivity, considering that the individual’s emotional reactivity decreases in cases where forgiveness is experienced in the individual. In other words, it can be concluded that two variables are predictors of each other. Therefore, it can be said that the findings obtained within the scope of the research support the literature data.

There is no study in which the concepts of forgiveness and emotional reactivity have been studied together. Therefore, understanding the dimension of the relationship between forgiveness-self-compassion and self-compassion-emotional reactivity can provide information about the causality of the relationship between forgiveness-emotional reactivity. The self-compassionate individual tends to forgive other people. Depending on this situation, the individual has a lower perception of threat and level of negative emotions (Neff & Pommier, 2013; Neff et al., 2007) because the feeling of threat is a cause of negative emotions such as anxiety or fear (Folkman, 2008). Negative emotions are important triggers of emotional reactivity (Berry et al., 2005; Edmondson, 2004). According to the findings of the study of Wenzel et al. (2010), it shows that forgiving a criminal makes it easier to evaluate the event in a calmer mood. Baker and McNulty (2011) stated that self-compassion has adverse and positive effects. According to these researchers, self-compassion can help individuals stay away from negative emotions. On the other hand, it can reduce the individual’s motivation in correcting the problems in relationships in the individual. This situation may cause negativity in the individual’s forgiveness behavior.

According to one of the another outcomes of the study was the relationship between forgiveness and happiness. Findings showed that there is a relationship between forgiveness and happiness. There are many studies in the literature in which the findings obtained in this study can be compared. García-Vázquez et al. (2020), Adam Karduz and Saricam (2018), Batık et al. (2017), Rana et al. (2014), and Maltby et al. (2005) found results supporting this finding in their studies. Since the rate of happiness increases in situations where forgiveness is experienced, it can be expressed that there is a positive correlation between forgiveness and happiness. In other words, it can be concluded that two variables are predictors of each other. Therefore, it can be said that the findings obtained within the scope of the research support the literature data.

One of the therapeutic tools that counselors who try to help a person overcome a person’s traumatic experience is to forgive those around the person and themselves (Cuğ, 2015). The individual ceases to harm the other person with forgiveness, and therefore the forgiving individual does not reflect on the injustices they have experienced in a deep context. Since they do not experience these thoughts deeply, their thoughts and evaluations about their own life become more positive. They communicate more positively with other people with whom they interact. The person feels that they belong to that society. As a result of this situation, the individuals evaluate themselves more healthily and increases subjective levels of well-being (Thompson et al., 2005; Tsang et al., 2006). Individuals with high levels of subjective well-being feel more positive emotions such as hope and love in their daily lives and feel fewer negative emotions such as anger and anxiety, and such individuals consider themselves more happy individuals (Cheavens et al., 2016; Sahranç et al., 2017; Zhang et al., 2015).

According to another outcome of the study, there is a significantly negative relationship between emotional reactivity and happiness. In other words, individuals who feel unhappy show more emotional reactions. Similar studies are seen on a limited number of subjects when the literature is examined. Therefore, there is limited data accumulation where research results can be compared.

Emotional reactivity is an individual’s reactions to emotions that emerge from relationships with other individuals throughout life (Yurdakul & Üner, 2015). For this reason, experiencing negative emotions leads to the development of negative emotional reactivity. In this context, these emotional reactions that occur in the individual may cause psychopathic problems. The individual may become depressed, and the individual will decrease the satisfaction of the individual and cause the individual to be unhappy (Cavanagh, et al., 2003). It can be said that emotional reactivity is one factor that negatively affects an individual’s happiness.

Another outcome of the study is that emotional reactivity mediates the relationship between forgiveness and happiness, according to the proposed model. As the individual’s forgiveness increases, his emotional reactivity decreases, and as the emotional reactivity decreases, the individual’s level of happiness increases.

An individuals with a high level of emotional reactivity cannot react to a suitable situation because they are too much under the influence of their emotions, cannot behave positively, and cannot develop positive relationships with other individuals around them. Since individuals cannot develop positive communication with the people around them, the individuals cannot develop self-compassion towards other individuals. Consequently, a decrease in forgiveness will be observed (Neff & Pommier, 2013; Wang et al., 2012). On the other hand, the individual cannot realize the opportunities, positive relationships, and potential around them. Because they cannot engage in positive behavior in situations that he/she encounters, and therefore cannot be happy because they cannot achieve a balance of emotions and thoughts (Licht & Chabot, 2006). The individuals, who can control their emotional reactions, become aware of their own positive and negative aspects, accept themselves as they are, and live life suitable for those factors, knowing the factors that will make them happy. In this context, it is expected that emotional reactivity has a mediating effect on the relationship between forgiveness and happiness.

There are no studies that examine the mediating role of emotional security and emotional reactivity between forgiveness and happiness that constitute the final output of the study. Therefore, there is no data to compare the research results directly. However, a literature review was conducted in which the variables of the research were examined and the results of the research were discussed in this context.

Studies show that forgiveness reduces negative emotions (anxiety, depression) and is an important positive predictor of individuals’ happiness level (Datu, 2014; Ramírez et al., 2014). Therefore, there is a positive and significant relationship between forgiveness and happiness. As a result, the results of the literature support the outcome of the research. However, according to the output results of the mediating role of emotional reactivity and emotional security in the relationship between forgiveness and happiness, which is the original aspect of the research. It was determined that emotional security contributed significantly to the positive meaningful relationship between forgiveness and happiness, as well as positive significant relationships among emotional security, forgiveness and happiness. Grych and Fincham (2001) state that individuals with low emotional security (exposed to destructive family conflicts) show symptoms of depression and anxiety. In this context, considering that negative experiences such as depression and anxiety negatively affect the happiness level of individuals (Ramírez et al., 2014), it can be inferred that emotional security can positively affect the level of happiness. According to the literature, there is a relationship between secure attachment and emotional security. (Davies & Cummings, 1998). Considering that there are positive significant relationships between secure attachment and forgiveness (Eraslan Çapan, 2018), the positive significant relationship between emotional security and forgiveness, which is one of the research results, is parallel to the literature results. According to the emotional security theory, children who do not feel safe may show psychopathological symptoms (Cummings & Davies, 2010). In this context, considering the negative correlations between psychopathology and forgiveness (Lee, 2011), there is a positive relationship between emotional security and forgiveness. Therefore, the research findings support the literature results.

Considering the positive correlations between emotional reactivity and psychopathology and the negative correlations between happiness and psychopathology, there is a negative correlation between emotional reactivity and happiness. Therefore, research outputs and literature results support the same context and contain similar results. In addition, when considered in the context of positive relationships between emotional reactivity and psychopathology, and negative relationships between forgiveness and psychological symptoms (childhood trauma, anxiety, bipolar disorder, depression) (Friedberg et al., 2009; Hirsch et al., 2012) it is possible to conclude that there is a negative correlation between emotional reactivity and forgiveness. Therefore, it can be said that the results of the research support the literature.

### Limitations and Future Research

The limitation of the study is related to the data collected and the method used. The data were collected using convenience sampling in a cross-sectional data design. The data obtained in the research is limited to the data collection tools which is used. The strengths of this study are that it has a theoretical framework, and a similar study has not been done before.

The model proposed in the present study can be used as a new model for researchers. The researchers and practitioners can prepare wellness psychoeducation programs for individuals in the university sample. This research was prepared by collecting data from students enrolled at universities in Turkey. The literature can be enriched by collecting data from different age groups and using different methods such as experimental study and Online Photovoice (OPV). Since this study was conducted in Turkey, it is limited to generalizing the results to Turkey. At this point, it would be beneficial to conduct similar studies with larger samples in different countries. It is thought that this present study provides the source for future studies.

## Financial Disclosure

The authors declare that this study received no financial support.

## Data Availability

The datasets generated during and/or analysed during the current study are available from the corresponding author on reasonable request.

## References

[CR1] Aki, B. D., Lamptey, E., Hembah, S. N., Oibiokpa, O. M., & Tachin, T. R. (2020). Covid-19 lockdown: psychological ımplications on life quality. *Journal of Human, Earth, and Future, 1*(2), 78-86. 10.28991/HEF-2020-01-02-04

[CR2] Albayrak, A. S. (2005). Çoklu doğrusal bağlantı halinde en küçük kareler tekniğinin alternatifi yanlı tahmin teknikleri ve bir uygulama. *ZKÜ Sosyal Bilimler Dergisi, 1*(1), 105-126. https://dergipark.org.tr/tr/pub/ijmeb/issue/54840/750869

[CR3] Arfasa, A. J., & Weldmeskel, F. M. (2020). Practices and challenges of guidance and counseling services in secondary schools. *Emerging Science Journal, 4*(3), 183-191. 10.28991/esj-2020-01222

[CR4] Arnett JJ (2000). Emerging adulthood: A theory of development from the late teens through the twenties. American Psychologist.

[CR5] Babin BJ, Zhuang W, Borges A (2021). Managing service recovery experience: effects of the forgiveness for older consumers. Journal of Retailing and Consumer Services.

[CR6] Baker LR, McNulty JK (2011). Self-compassion and relationship maintenance: The moderating roles of conscientiousness and gender. Journal of Personality and Social Psychology.

[CR7] Bartle-Haring S, Rosen KH, Stith SM (2002). Emotional reactivity and psychological distress. Journal of Adolescent Research.

[CR8] Batık MV, Bingöl TY, Kodaz AF, Hoşoğlu R (2017). Forgiveness and subjective happiness of university students. International Journal of Higher Education.

[CR9] Belicki K, DeCourville N, Kamble SV, Stewart T, Rubel A (2020). Reasons for forgiving: Individual differences and emotional outcomes. SAGE Open.

[CR10] Berenbaum H, Williams M (1995). Personality and emotional reactivity. Journal of Research in Personality.

[CR11] Bergman KN, Cummings EM, Davies PT (2014). Interparental aggression and adolescent adjustment: The role of emotional insecurity and adrenocortical activity. Journal of Family Violence.

[CR12] Berry JW, Worthington EL, O’Connor LE, Parrott L, Wade NG (2005). Forgivingness, vengeful rumination, and affective traits. Journal of Personality.

[CR13] Black A.L. (2020) Picturing childhood connections: How arts-based reflection and representation strengthen preservice early childhood teachers’ understandings about well-being, belonging, and place. In L. McKay, G. Barton, S. Garvis, & V. Sappa (Eds.), A*rts-Based Research, Resilience and Well-being Across the Lifespan.* Palgrave Macmillan. 10.1007/978-3-030-26053-8_17

[CR14] Bono G, McCullough ME, Root LM (2008). Forgiveness, feeling connected to others, and well-being: Two longitudinal studies. Personality and Social Psychology Bulletin.

[CR15] Bowlby, J. (1951). *Maternal care and mental health* (Vol. 2). WHO. PMC2554008 14821768

[CR16] Bronfenbrenner U (1986). Ecology of the family as a context for human development: Research perspectives. Developmental Psychology.

[CR17] Brülde B (2007). Happiness theories of the good life. Journal of Happiness Studies.

[CR18] Buehler C, Welsh DP (2009). A process model of adolescents' triangulation into parents' marital conflict: the role of emotional reactivity. Journal of Family Psychology.

[CR19] Bugay A, Demir A (2010). A Turkish version of heartland forgiveness scale. Procedia - Social and Behavioral Sciences.

[CR20] Büyüköztürk, Ş., Kılıç Çakmak, E., Akgün, Ö. E., Karadeniz, Ş., & Demirel, F. (2014). *Bilimsel araştırma yöntemleri.* Ankara.

[CR21] Bylsma LM, Taylor-Clift A, Rottenberg J (2011). Emotional reactivity to daily events in major and minor depression. Journal of Abnormal Psychology.

[CR22] Cantón-Cortés D, Cortés MR, Cantón J (2019). Pathways from childhood sexual abuse to trait anxiety. Child Abuse & Neglect.

[CR23] Cantón-Cortés D, Cortés MR, Cantón J (2020). Child sexual abuse and suicidal ideation: The differential role of attachment and emotional security in the family system. International Journal of Environmental Research and Public Health.

[CR24] Carthy T, Horesh N, Apter A, Gross JJ (2010). Patterns of emotional reactivity and regulation in children with anxiety disorders. Journal of Psychopathology and Behavioral Assessment.

[CR25] Cavanagh JT, Carson AJ, Sharpe M, Lawrie SM (2003). Psychological autopsy studies of suicide: A systematic review. Psychological Medicine.

[CR26] Chagigiorgis H, Paivio S, Malcolm W, DeCourville N, Belicki K (2008). Forgiveness as an outcome in emotion-focused trauma therapy. Women's reflections on the complexities of forgiveness.

[CR27] Cheavens JS, Cukrowicz KC, Hansen R, Mitchell SM (2016). Incorporating resilience factors into the interpersonal theory of suicide: The role of hope and self-forgiveness in an older adult sample. Journal of Clinical Psychology.

[CR28] Cheung RYM (2020). Constructive interparental conflict and child adjustment in the chinese context: A moderated mediation model of emotional security and disintegration avoidance. Journal of Child and Family Studies.

[CR29] Chung JWY, So HCF, Choi MMT, Yan VCM, Wong TKS (2021). Artificial Intelligence in education: Using heart rate variability (HRV) as a biomarker to assess emotions objectively. Computers and Education: Artificial Intelligence.

[CR30] Cleare S, Gumley A, O'Connor RC (2019). Self-compassion, self-forgiveness, suicidal ideation, and self-harm: A systematic review. Clinical Psychology & Psychotherapy.

[CR31] Commodari E (2013). Preschool teacher attachment, school readiness and risk of learning difficulties. Early Childhood Research Quarterly.

[CR32] Cromwell HC, Abe N, Barrett KC, Caldwell-Harris C, Gendolla GH, Koncz R, Sachdev PS (2020). Mapping the interconnected neural systems underlying motivation and emotion: A key step toward understanding the human affectome. Neuroscience & Biobehavioral Reviews.

[CR33] Cuğ, F. D. (2015). *Self-forgiveness, self-compassion, subjective vitality, and orientation to happiness as predictors of subjective well-being* (Unpublished doctoral dissertation). Middle East Technical University.

[CR34] Cummings EM, Cheung RYM, Davies PT (2013). Prospective relations between parental depression, negative expressiveness, emotional insecurity, and children’s ınternalizing symptoms. Child Psychiatry & Human Development.

[CR35] Cummings EM, Davies PT (2010). Marital conflict and children: An emotional security perspective.

[CR36] Dansby Olufowote RA, Fife ST, Schleiden C, Whiting JB (2019). How Can I Become More Secure?: A Grounded Theory of Earning Secure Attachment. Journal of Marital and Family Therapy.

[CR37] Datu JAD (2014). Forgiveness, gratitude and subjective well-being among Filipino adolescents. International Journal for the Advancement of Counselling.

[CR38] Davidson RJ (1998). Affective style and affective disorders: Perspectives from affective neuroscience. Cognition & Emotion.

[CR39] Davies PT, Cummings EM (1998). Exploring children’s emotional security as a mediator of the link between marital relations and child adjustment. Child Development.

[CR40] Davies PT, Cummings EM (1994). Marital conflict and child adjustment: An emotional security hypothesis. Psychological Bulletin.

[CR41] Davies PT, Woitach MJ (2008). Children's emotional security in the interparental relationship. Current Directions in Psychological Science.

[CR42] Davies PT, Forman EM, Rasi JA, Stevens KI (2002). Assessing children's emotional security in the interparental relationship: The security in the interparental subsystem scales. Child Development.

[CR43] Davies PT, Parry LQ, Bascoe SM, Cicchetti D, Cummings EM (2020). Interparental conflict as a curvilinear risk factor of youth emotional and cortisol reactivity. Developmental Psychology.

[CR44] Delle Fave A, Brdar I, Freire T, Vella-Brodrick D, Wissing MP (2011). The eudaimonic and hedonic components of happiness: Qualitative and quantitative findings. Social Indicators Research.

[CR45] DeNeve KM, Cooper H (1998). The happy personality: A meta-analysis of 137 personality traits and subjective well-being. Psychological Bulletin.

[CR46] Diener E (2006). Guidelines for national indicators of subjective well-being and ill-being. Journal of Happiness Studies.

[CR47] Diener E, Seligman ME (2002). Very happy people. Psychological Science.

[CR48] Doğan, T., & Akıncı Çötok, N. (2011). Adaptation of the short form of the Oxford Happiness Questionnaire into Turkish: A validity and reliability study. *Türk Psikolojik Danışma ve Rehberlik Dergisi, 4*(36) , 165-170. https://dergipark.org.tr/tr/pub/tpdrd/issue/21456/229762

[CR49] Dorn, O., & Schudlich, T. D. D. R. (2020). Enduring effects of infant emotional security on preschooler adaptation to interparental conflict. *In* *Parenting*. IntechOpen. 10.5772/intechopen.91261

[CR50] Edmondson AC, Kramer RM, Cook KS (2004). Psychological Safety, Trust, and Learning in Organizations: A Group-Level Lens. Trust and distrust in organizations: Dilemmas and approaches.

[CR51] Eisenberg N, Duckworth AL, Spinrad TL, Valiente C (2014). Conscientiousness: Origins in childhood?. Developmental Psychology.

[CR52] El-Sheikh M, Buckhalt JA, Mark Cummings E, Keller P (2007). Sleep disruptions and emotional insecurity are pathways of risk for children. Journal of Child Psychology and Psychiatry.

[CR53] Eraslan Çapan, B. (2018). Secure attachment and forgiveness: the mediating role of psychological resilience. *Hacettepe Üniversitesi Eğitim Fakültesi Dergisi (H. U. Journal of Education) 33*(2): 280-292. doi:10.16986/HUJE.2017030459.

[CR54] Ercengiz, M. (2019). Ergenlerde duygu düzenleme stratejilerinin yordayıcı olarak mutluluk düzeyindeki rolü*. Ekev Akademi Dergisi, 23* (77), 267-278. http://www.ekevakademi.org/Makaleler/1547094369_17%20Mustafa%20ERCENGIZ.pdf

[CR55] Ermer AE, Proulx CM (2016). Unforgiveness, depression, and health in later life: The protective factor of forgivingness. Aging & Mental Health.

[CR56] Fabbri-Destro M, Rizzolatti G (2008). Mirror neurons and mirror systems in monkeys and humans. Physiology.

[CR57] Fernandes, H. I. V. M., Andrade, L. M. C., Martins, M. M., Rolim, K. M. C., Millions, R. M., Frota, M. A., & Albuquerque, F. H. S. (2020). Happiness as a strength in the promotion of adolescent and adult young health. *Revista Brasileira de Enfermagem*, *73*(3). 10.1590/0034-7167-2019-0064 10.1590/0034-7167-2019-0064 32321141

[CR58] Fincham FD, Beach SR, Davila J (2007). Longitudinal relations between forgiveness and conflict resolution in marriage. Journal of Family Psychology.

[CR59] Fletcher, J. M., & Schurer, S. (2017). Origins of adulthood personality: The role of adverse childhood experiences. *The B.E. Journal of Economic Analysis & Policy, 17*(2). 10.1515/bejeap-2015-0212 10.1515/bejeap-2015-0212 PMC6063370 30057657

[CR60] Folkman S (2008). The case for positive emotions in the stress process. Anxiety, Stress, and Coping.

[CR61] Ford TE, Lappi SK, Holden CJ (2016). Personality, humor styles and happiness: Happy people have positive humor styles. Europe's Journal of Psychology.

[CR62] Fox E, Cahill S, Zougkou K (2010). Preconscious processing biases predict emotional reactivity to stress. Biological Psychiatry.

[CR63] Friedberg JP, Suchday S, Srinivas VS (2009). Relationship between forgiveness and psychological and physiological indices in cardiac patients. International Journal of Behavioral Medicine.

[CR64] Gall TL, Bilodeau C (2020). The role of forgiveness as a coping response to intimate partner stress. Journal of Spirituality in Mental Health.

[CR65] García-Vázquez FI, Valdés-Cuervo AA, Martínez-Ferrer B, Parra-Pérez LG (2020). Forgiveness, gratitude, happiness, and prosocial bystander behavior in bullying. Frontiers in Psychology.

[CR66] Girme YU, Jones RE, Fleck C, Simpson JA, Overall NC (2020). Infants’ attachment insecurity predicts attachment-relevant emotion regulation strategies in adulthood. Emotion.

[CR67] Goeke-Morey MC, Cummings EM, Papp LM (2007). Children and marital conflict resolution: Implications for emotional security and adjustment. Journal of Family Psychology.

[CR68] Gosling CJ, Caparos S, Moutier S (2020). The interplay between the importance of a decision and emotion in decision-making. Cognition and Emotion.

[CR69] Gottlieb G, Mussen PM (1983). The psychobiological approach to developmental issues. Handbook of child psychology.

[CR70] Graham C (2008). Happiness and health: Lessons—and questions—for public policy. Health Affairs.

[CR71] Griffin, B. J., Worthington, E. L., Jr., Lavelock, C. R., Wade, N. G., & Hoyt, W. T. (2015). Forgiveness and mental health. In L. L. Toussaint, E. L. Worthington, Jr., & D. R. Williams (Eds.), *Forgiveness and health: Scientific evidence and theories relating forgiveness to better health, *pp. 77-90. Springer Science + Business Media. 10.1007/978-94-017-9993-5_6

[CR72] Gross, J. J., & Thompson, R. A. (2007). *Emotion regulation: Conceptual foundations*. Guilford.

[CR73] Gross JT, Stern JA, Brett BE, Cassidy J (2017). The multifaceted nature of prosocial behavior in children: Links with attachment theory and research. Social Development.

[CR74] Gruber J, Mauss IB, Tamir M (2011). A dark side of happiness? How, when, and why happiness is not always good. Perspectives on Psychological Science.

[CR75] Hayes AF (2017). Introduction to mediation, moderation, and conditional process analysis: A regression-based approach.

[CR76] Hill J, Healey RL, West H, Déry C (2021). Pedagogic partnership in higher education: encountering emotion in learning and enhancing student wellbeing. Journal of Geography in Higher Education.

[CR77] Hills P, Argyle M (2002). The Oxford Happiness Questionnaire: A compact scale for the measurement of psychological well-being. Personality and Individual Differences.

[CR78] Hirsch JK, Webb JR, Jeglic EL (2012). Forgiveness as a moderator of the association between anger expression and suicidal behavior. Ment Health Relig Cul.

[CR79] Hoffman K, Cooper G, Powell B (2017). Raising a secure child: how circle of security parenting can help you nurture your child's attachment, emotional resilience, and freedom to explore.

[CR80] Hoyniak, C. P., Bates, J. E., McQuillan, M. E., Albert, L. E., Staples, A. D., Molfese, V. J., & Deater-Deckard, K. (2020). The family context of toddler sleep: routines, sleep environment, and emotional security ınduction in the hour before bedtime. *Behavioral Sleep Medicine,**19*(6), 795–813. 10.1080/15402002.2020.1865356 10.1080/15402002.2020.1865356 PMC8233403 33356565

[CR81] Immordino-Yang MH, Yang XF, Damasio H (2016). Cultural modes of expressing emotions influence how emotions are experienced. Emotion.

[CR82] Johnson HD, Wernli MA, LaVoie JC (2013). Situational, interpersonal, and intrapersonal characteristic associations with adolescent conflict forgiveness. The Journal of Genetic Psychology.

[CR83] Joshanloo M (2014). Eastern conceptualizations of happiness: Fundamental differences with western views. Journal of Happiness Studies.

[CR84] Adam Karduz, F.F., & Saricam, H. (2018). The Relationships between positivity, forgiveness, happiness, and revenge. *Revista Romaneasca pentru Educatie Multidimensionala, 10*(4), 1-22. 10.18662/rrem/68

[CR85] Karremans JC, Van Lange PA, Holland RW (2005). Forgiveness and its associations with prosocial thinking, feeling, and doing beyond the relationship with the offender. Personality and Social Psychology Bulletin.

[CR86] King LA, Napa CK (1998). What makes a life good?. Journal of Personality and Social Psychology.

[CR87] Knutson J, Enright R, Garbers B (2008). Validating the developmental pathway of forgiveness. Journal of Counseling & Development.

[CR88] Konjedi S, Maleeh R (2017). A closer look at the relationship between the default network, mind wandering, negative mood, and depression. Cognitive, Affective, & Behavioral Neuroscience.

[CR89] La Fors K (2020). Legal remedies for a forgiving society: Children's rights, data protection rights and the value of forgiveness in AI-mediated risk profiling of children by Dutch authorities. Computer Law & Security Review.

[CR90] Lawler-Row KA, Younger JW, Piferi RL, Jones WH (2006). The role of adult attachment style in forgiveness following an interpersonal offense. Journal of Counseling & Development.

[CR91] Leary, M., Tate, E., Adams, C., Allen, A., & Hancock, J. (2007). Self-compassion and reactions to unpleasant self-relevant events: The implications of treating oneself kindly. *Journal of Personality and Social Psychology, 92*, 887–904. 10. 1037/0022-3514.92.5.887. 10.1037/0022-3514.92.5.887 17484611

[CR92] Lee, Y.R. (2011). *Forgiveness education for women with fibromyalgia who have been abused in childhood by their parents*, Doctoral Dissertation, University of Wisconsin, Madison.

[CR93] Li Z, Sturge-Apple ML, Liu S, Davies PT (2020). Parent-adolescent physiological synchrony: Moderating effects of adolescent emotional insecurity. Psychophysiology.

[CR94] Licht C, Chabot D (2006). The Chabot emotional differentiation scale: A theoretically and psychometrically sound instrument for measuring Bowen's intrapsychic aspect of differentiation. Journal of Marital and Family Therapy.

[CR95] Liese BS, Kim HS, Hodgins DC (2020). Insecure attachment and addiction: Testing the mediating role of emotion dysregulation in four potentially addictive behaviors. Addictive Behaviors.

[CR96] Liu Y, Li H, Xu X, Li Y, Wang Z, Zhu H, Zhang X, Jiang S, Li N, Gu S, Wang F, Huang JH (2020). The relationship between insecure attachment to depression: Mediating role of sleep and cognitive reappraisal. Neural Plasticity.

[CR97] Long KN, Chen Y, Potts M, Hanson J, VanderWeele TJ (2020). Spiritually motivated self-forgiveness and divine forgiveness, and subsequent health and well-being among middle-aged female nurses: an outcome-wide longitudinal approach. Frontiers in Psychology.

[CR98] Lozano M, Solé-Auró A (2021). Happiness and life expectancy by main occupational position among older workers: Who will live longer and happy?. SSM - Population Health.

[CR99] Lucas RE, Baird BM (2004). Extraversion and emotional reactivity. Journal of Personality and Social Psychology.

[CR100] Maltby, J., Day, L., & Barber, L. (2005). Forgiveness and happiness. The differing contexts of forgiveness using the distinction between hedonic and eudaimonic happiness. *Journal of Happiness Studies*, *6*(1), 1-13. 10.1007/s10902-004-0924-9

[CR101] Mansfield CD, Pasupathi M, McLean KC (2015). Is narrating growth in stories of personal transgressions associated with increased well-being, self-compassion, and forgiveness of others?. Journal of Research in Personality.

[CR102] Maremmani, A. G. I., Maiello, M., Carbone, M. G., Pallucchini, A., Brizzi, F., Belcari, I., & Maremmani, I. (2018). Towards a psychopathology specific to Substance Use Disorder: Should emotional responses to life events be included? *Comprehensive Psychiatry,**80*, 132–139. 10.1016/j.comppsych.2017.10.001 10.1016/j.comppsych.2017.10.001 29091779

[CR103] Masterson JF (2008). Bağlanma kuramı ve nörobiyolojik kendilik gelişimi açısından kişilik bozuklukları.

[CR104] McCullough ME (2000). Forgiveness as human strength: Theory, measurement, and links to well-being. Journal of Social and Clinical Psychology.

[CR105] McCullough ME, Rachal KC, Sandage SJ, Worthington EL, Brown SW, Hight TL (1998). Interpersonal forgiving in close relationships: II. Theoretical elaboration and measurement. Journal of Personality and Social Psychology.

[CR106] McCullough ME, Worthington EL, Rachal KC (1997). Interpersonal forgiving in close relationships. Journal of Personality and Social Psychology.

[CR107] McLaughlin, K. A., Fairbank, J. A., Gruber, M. J., Jones, R. T., Osofsky, J. D., Pfefferbaum, B., & Kessler, R. C. (2010). Trends in serious emotional disturbance among youths exposed to hurricane katrina. *Journal of the American Academy of Child & Adolescent Psychiatry,**49*(10), 990–1000. 10.1016/j.jaac.2010.06.012 10.1016/j.jaac.2010.06.012 PMC3228600 20855044

[CR108] Michalos AC (2008). Education, happiness and wellbeing. Social Indicators Research.

[CR109] Millgram Y, Huppert JD, Tamir M (2020). Emotion goals in psychopathology: A new perspective on dysfunctional emotion regulation. Current Directions in Psychological Science.

[CR110] Montijn ND, Gerritsen L, Engelhard IM (2021). Forgetting the future: Emotion improves memory for imagined future events in healthy individuals but not individuals with anxiety. Psychological Science.

[CR111] Mousavi Asl E, Abdi L, Sadegh AM, Behrouzian F (2021). The mediating role of self-compassion in the relationship between positive reactivity, negative reactivity, and perfectionism with disordered eating. Journal of Education and Health Promotion.

[CR112] Nagra GS, Lin A, Upthegrove R (2016). What bridges the gap between self-harm and suicidality? The role of forgiveness, resilience and attachment. Psychiatry Research.

[CR113] Nandrino JL, Dodin V, Cottencin O, Doba K (2020). Effect of intrapersonal emotional competences on the relationship between attachment insecurity and severity of eating disorder symptoms in patients with restrictive anorexia. Journal of Clinical Psychology.

[CR114] Neff KD, Pommier E (2013). The relationship between self-compassion and other-focused concern among college undergraduates, community adults, and practicing meditators. Self and Identity.

[CR115] Neff KD, Rude SS, Kirkpatrick KL (2007). An examination of self compassion in relation to positive psychological functioning and personality traits. Journal of Research in Personality.

[CR116] Niklas F, Cohrssen C, Tayler C (2016). Improving preschoolers’ numerical abilities by enhancing the home numeracy environment. Early Education and Development.

[CR117] Nock MK, Wedig MM, Holmberg EB, Hooley JM (2008). The emotion reactivity scale: Development, evaluation, and relation to self-injurious thoughts and behaviors. Behavior Therapy.

[CR118] O’Neill J (2008). Happiness and the good life. Environmental Values.

[CR119] Oti-Boadi M, Dankyi E, Kwakye-Nuako CO (2020). Stigma and forgiveness in ghanaian mothers of children with autism spectrum disorders (ASD). Journal of Autism and Developmental Disorders.

[CR120] Pan Z, Zhang D, Hu T, Pan Y (2018). The relationship between psychological Suzhi and social anxiety among Chinese adolescents: the mediating role of self-esteem and sense of security. Child and Adolescent Psychiatry and Mental Health.

[CR121] Park S, Kim SA, Park WS (2017). Relationship between health behavior and subjective unhappiness in high school students. Journal of Agricultural Medicine and Community Health.

[CR122] Pelechano V, González-Leandro P, García L, Morán C (2013). Is it possible to be too happy? Happiness, personality, and psychopathology. International Journal of Clinical and Health Psychology.

[CR123] Ponce-Pardo, A., Acosta-Rodas, P., Cruz-Cárdenas, J., & Ramos-Galarza, C. (2021). Music stimulation as a method of optimizing autobiographical memory in patients diagnosed with alzheimer’s disease. *Emerging Science Journal, 5*(5), 678-687. 10.28991/esj-2021-01304

[CR124] Ramírez, E., Ortega, A. R., Chamorro, A., & Colmene-ro, J. M. (2014). A program of positive intervention in the elderly: Memories, gratitude and forgiveness. *Aging & Mental Health,18*, 463–470. doı: 10.1080/13607863.2013.856858 10.1080/13607863.2013.856858 24229346

[CR125] Rana S, Hariharan M, Nandinee D, Vincent K (2014). Forgiveness: A determinant of adolescents’ happiness. Indian Journal of Positive Psychology.

[CR126] Ripper CA, Boyes ME, Clarke PJ, Hasking PA (2018). Emotional reactivity, intensity, and perseveration: Independent dimensions of trait affect and associations with depression, anxiety, and stress symptoms. Personality and Individual Differences.

[CR127] Rodriguez ET, Tamis-LeMonda CS (2011). Trajectories of the home learning environment across the first 5 years: Associations with children’s vocabulary and literacy skills at prekindergarten. Child Development.

[CR128] Russell, L. (2020). The who, the what, and the how of forgiveness. *Philosophy Compass, 15*(3). 10.1111/phc3.12656

[CR129] Ryan RM, Deci EL (2001). On happiness and human potentials: A review of research on hedonic and eudaimonic well-being. Annual Review of Psychology.

[CR130] Sahranç Ü, Çelik E, Turan ME (2017). Mediating and moderating effects of social support in the relationship between social anxiety and hope levels in children. Journal of Happiness Studies.

[CR131] Sakız, E. (2011). *Duygusal güvenlik hipotezi, ebeveyn tutumları, evlilik çatışması ve çocuk uyum problemleri arasındaki ilişkilerin incelenmesi* (Unpublished master’s thesis). Istanbul University.

[CR132] Santos M, Egerstedt M (2020). From motions to emotions: Can the fundamental emotions be expressed in a robot swarm?. International Journal of Social Robotics.

[CR133] Scott, J., Kallestad, H., Vedaa, O., Sivertsen, B., & Etain, B. (2021). Sleep disturbances and first onset of major mental disorders in adolescence and early adulthood: a systematic review and meta-analysis. *Sleep Medicine Reviews,**101429*,. 10.1016/j.smrv.2021.101429 10.1016/j.smrv.2021.101429 33549912

[CR134] Seçer, İ., Halmatov, S., & Gençdoğan, B. (2013). Duygusal tepkisellik ölçeği’nin türkçeye uyarlanması: güvenirlik ve geçerlilik çalışması. *Sakarya University Journal of Education,* 3(1), 77-89. https://dergipark.org.tr/tr/download/article-file/192292

[CR135] Seligman MEP, Snyder CR, Lopez SJ (2002). Positive psychology, positive prevention, and positive therapy. Handbook of positive psychology.

[CR136] Shablack H, Becker M, Lindquist KA (2020). How do children learn novel emotion words? A study of emotion concept acquisition in preschoolers. Journal of Experimental Psychology: General.

[CR137] Shapero BG, Farabaugh A, Terechina O, DeCross S, Cheung JC, Fava M, Holt DJ (2019). Understanding the effects of emotional reactivity on depression and suicidal thoughts and behaviors: Moderating effects of childhood adversity and resilience. Journal of Affective Disorders.

[CR138] Shoshani A, Braverman S, Meirow G (2021). Video games and close relations: Attachment and empathy as predictors of children's and adolescents' video game social play and socio-emotional functioning. Computers in Human Behavior.

[CR139] Silk JS, Steinberg L, Morris AS (2003). Adolescents' emotion regulation in daily life: Links to depressive symptoms and problem behavior. Child Development.

[CR140] Silva CS, Calheiros MM, Carvalho H (2016). Interparental conflict and adolescents’ self-representations: The role of emotional insecurity. Journal of Adolescence.

[CR141] Singer T, Lamm C (2009). The social neuroscience of empathy. Annals of the New York Academy of Sciences.

[CR142] Snefjella B, Lana N, Kuperman V (2020). How emotion is learned: Semantic learning of novel words in emotional contexts. Journal of Memory and Language.

[CR143] Sudirman, S. A., Suud, F. M., Rouzi, K. S., & Sari, D. P. (2019). Forgiveness and happiness through resilience. *Jurnal Al Qalb, 2*(10), 113-132. 10.15548/alqalb.v10i2.955

[CR144] Şendil, G. (2016). Duygusal Güvenlik Ölçeği (DGÖ): Üniversite öğrencilerinde geçerlik ve güvenirlik çalışması. *Psikoloji Çalışmaları Dergisi, 36*(2), 45 - 64. https://dergipark.org.tr/tr/pub/iupcd/issue/28559/304768

[CR145] Tanhan A (2020). Utilizing Online Photovoice (OPV) methodology to address biopsychosocial spiritual economic issues and wellbeing during COVID-19: Adapting OPV to Turkish. Turkish Studies.

[CR146] Tanhan, A., Arslan, G., Yavuz, K. F., Young, J. S., Çiçek, İ., Akkurt, M. N., Ulus, İ. Ç., Görünmek, E. T., Demir, R., Kürker, F., Çelik, C., Akça, M. Ş., Ünverdi, B., Ertürk, H., & Allen, K. (2021). A constructive understanding of mental health facilitators and barriers through Online Photovoice (OPV) during COVID-19. *ESAM Ekonomik ve Sosyal Araştırmalar Dergisi, 2*(2), 214-249. https://dergipark.org.tr/en/pub/esamdergisi/issue/64932/956618

[CR147] Tekinalp, B. E., & Terzi, Ş. (2012). Terapötik bir araç olarak bağışlama: iyileştirici etken olarak bağışlama olgusunun psikolojik danışma sürecinde kullanımı. *Eğitim ve Bilim*, *37*(166), 14-24. http://egitimvebilim.ted.org.tr/index.php/EB/article/view/405

[CR148] Thompson, L. Y., Snyder, C. R., Hoffman, L., Michael, S. T., Rasmussen, H. N., Billings, L. S., & Roberts, D. E. (2005). Dispositional forgiveness of self, others, and situations. *Journal of Personality,**73*(2), 313–360. 10.1111/j.1467-6494.2005.00311.x 10.1111/j.1467-6494.2005.00311.x 15745433

[CR149] Tsang J-A, McCullough ME, Fincham FD (2006). The longitudinal association between forgiveness and relationship closeness and commitment. Journal of Social and Clinical Psychology.

[CR150] Turan, M. E. (2021). The relationship between social emotional learning competencies and life satisfaction in adolescents: Mediating role of academic resilience. *International Online Journal of Educational Sciences, 13*(4), 1126-1142. 10.15345/iojes.2021.04.012

[CR151] Uddin J, Alharbi N, Uddin H, Hossain MB, Hatipoğlu SS, Long DL, Carson AP (2020). Parenting stress and family resilience affect the association of adverse childhood experiences with children's mental health and attention-deficit/hyperactivity disorder. Journal of Affective Disorders.

[CR152] Van der Wal RC, Karremans JC, Cillessen AHN (2017). Causes and consequences of children's forgiveness. Child Development Perspectives.

[CR153] Viner RM, Ross D, Hardy R, Kuh D, Power C, Johnson A, Batty GD (2015). Life course epidemiology: Recognising the importance of adolescence. Journal of Epidemiology and Community Health.

[CR154] Wang CD, King ML, Debernardi NR (2012). Adult attachment, cognitive appraisal, and university students’ reactions to romantic infidelity. Journal of College Counseling.

[CR155] Watkins DA, Hui EK, Luo W, Regmi M, Worthington EL, Hook JN, Davis DE (2011). Forgiveness and interpersonal relationships: A Nepalese investigation. The Journal of Social Psychology.

[CR156] Wenzel M, Turner JK, Okimoto TG (2010). Is forgiveness an outcome or initiator of sociocognitive processes? Rumination, empathy, and cognitive appraisals following a transgression. Social Psychological and Personality Science.

[CR157] Wilkowski BM, Robinson MD, Troop-Gordon W (2010). How does cognitive control reduce anger and aggression? The role of conflict monitoring and forgiveness processes. Journal of Personality and Social Psychology.

[CR158] Winpenny EM, Winkler MR, Stochl J, van Sluijs EMF, Larson N, Neumark-Sztainer D (2020). Associations of early adulthood life transitions with changes in fast food intake: A latent trajectory analysis. The International Journal of Behavioral Nutrition and Physical Activity.

[CR159] Worthington EL, vanOyen Witvliet C, Lerner AJ, Scherer M (2005). Forgiveness in health research and medical practice. Explore.

[CR160] Wu Q, Chi P, Zeng X (2019). Roles of anger and rumination in the relationship between self-compassion and forgiveness. Mindfulness.

[CR161] Yedirir, S., & Hamarta, E. (2015). Emotional expression and spousal support as predictors of marital satisfaction: The case of Turkey. *Educational Sciences: Theory and Practice*, *15*(6), 1549-1558. 10.12738/estp.2015.6.2822

[CR162] Yıldırım M, Güler A (2021). Positivity explains how COVID-19 perceived risk increases death distress and reduces happiness. Personality and Individual Differences.

[CR163] Yurdakul A, Üner S (2015). The evaluation of emotional reactivity status of school of health students. TAF Preventive Medicine Bulletin.

[CR164] Zafar H, Debowska A, Boduszek D (2021). Emotion regulation difficulties and psychopathology among Pakistani adolescents. Clinical Child Psychology and Psychiatry.

[CR165] Zhang Q, Ting-Toomey S, Oetzel JG, Zhang J (2015). The emotional side of forgiveness: A cross-cultural investigation of the role of anger and compassion and face threat in interpersonal forgiveness and reconciliation. Journal of International and Intercultural Communication.

